# Exploring the role of microglia in mood disorders associated with experimental multiple sclerosis

**DOI:** 10.3389/fncel.2015.00243

**Published:** 2015-06-25

**Authors:** Antonietta Gentile, Francesca De Vito, Diego Fresegna, Alessandra Musella, Fabio Buttari, Silvia Bullitta, Georgia Mandolesi, Diego Centonze

**Affiliations:** ^1^Fondazione Santa Lucia/Centro Europeo per la Ricerca sul Cervello (CERC)Rome, Italy; ^2^Clinica Neurologica, Dipartimento di Medicina dei Sistemi, Università Tor VergataRome, Italy; ^3^IRCCS Istituto Neurologico Mediterraneo (INM) NeuromedPozzilli, Italy

**Keywords:** microglia, experimental autoimmune encephalomyelitis, multiple sclerosis, behavioral syndrome, neuron-microglia interaction, depression, anxiety

## Abstract

Microglia is increasingly recognized to play a crucial role in the pathogenesis of psychiatric diseases. In particular, microglia may be the cellular link between inflammation and behavioral alterations: by releasing a number of soluble factors, among which pro-inflammatory cytokines, that can regulate synaptic activity, thereby leading to perturbation of behavior. In multiple sclerosis (MS), the most common neuroinflammatory disorder affecting young adults, microglia activation and dysfunction may account for mood symptoms, like depression and anxiety, that are often diagnosed in patients even in the absence of motor disability. Behavioral studies in experimental autoimmune encephalomyelitis (EAE), the animal model of MS, have shown that emotional changes occur early in the disease and in correlation to inflammatory mediator and neurotransmitter level alterations. However, such studies lack a full and comprehensive analysis of the role played by microglia in EAE-behavioral syndrome. We review the experimental studies addressing behavioral symptoms in EAE, and propose the study of neuron-glia interaction as a powerful but still poorly explored tool to investigate the burden of microglia in mood alterations associated to MS.

## Introduction

Multiple Sclerosis (MS) is a chronic inflammatory demyelinating and neurodegenerative disease of the central nervous system (CNS), which represents the leading cause of non-traumatic disability in young adults in Western countries (Compston and Coles, 2008). In addition to physical impairment, MS is frequently associated to mood disorders, like anxiety and depression even in non-disabled patients (Marrie et al., 2009). Structural and functional brain changes, induced by inflammation, seem to be implicated in MS psychiatric symptoms (Feinstein et al., 2014). In fact, T-lymphocytes peripherally primed against myelin-like components infiltrate the brain and initiate a chain of inflammatory events, including both gray and white matter microgliosis (Centonze et al., 2010). Interestingly, recent studies in MS animal model, the experimental autoimmune encephalomyelitis (EAE), have shown that activated microglia, by releasing pro-inflammatory cytokines, can alter brain synaptic transmission also before the appearance of motor symptoms (Centonze et al., 2009, 2010). Notably, changes in neuronal compartment and abnormalities in microglial physiology have been related to several psychopathological conditions in both humans and animal models (Price and Drevets, 2010; Frick et al., 2013), raising the possibility that microglia may have a crucial role in mediating mood disorders in MS.

This paper briefly discusses the current studies about anxious- and depressive-like behaviors in the EAE model and proposes the contribution of microglia to EAE mood disorders, as an important but quite unexplored field for future research.

## Microglia in Mood Disorders

Microglia derive from macrophage lineage and represent 10–20% of the glial cells in the CNS (Lynch, 2009). Exposure to foreign antigens or cellular debris induces microglial cytotoxic activation required for brain immune surveillance. Such activated microglia release a variety of proinflammatory mediators, like interleukin-1β (IL-1β) and tumor necrosis factor (TNF; Lynch, 2009; Kettenmann et al., 2011).

However, cytokines, such as interleukin-4 (IL-4) or interleukin-25 (IL-25), can shift microglial state from the resting to the neuroprotective one (Zhou et al., 2012; Maiorino et al., 2013). Activated microglia can switch from the characteristic ramified morphology to the hyperramified or amoeboid/phagocytic one with upregulation of several markers, including the ionized calcium-binding adapter molecule 1 (Iba1) and CD11b (Kettenmann et al., 2011).

Microglia mutually interact by contact or via mediators with several cells, like T cells, astrocytes and even neurons and such interactions can induce changes in microglial state (Lynch, 2009). The direct and indirect communication between microglia and neurons reveals microglia synaptic functions in addition to the well-established immune ones. In fact, it has been demonstrated that microglia participate in neurogenesis (Butovsky et al., 2006), neuronal transmission (Graeber, 2010) and synaptic pruning in memory and neuronal plasticity (Schafer et al., 2012).

Both abnormalities in microglial physiology and dysfunction of neuron-microglia cross-talk have been involved in psychiatric diseases. For example, several *postmortem* studies have reported abundant activated microglia surrounding neurons (Vargas et al., 2005) and a switch to amoeboid morphology (Morgan et al., 2010; Tetreault et al., 2012) in multiple brain regions from patient with autism spectrum disorders. Moreover, positron emission tomography (PET) imaging has confirmed *postmortem* findings about microglial activation in autism patients (Suzuki et al., 2013). Similar results have been observed in different animal models of autism (Zerrate et al., 2007; Heo et al., 2011; MacFabe et al., 2011). Furthermore, a recent work in MECP2-null mice, which model Rett syndrome, an X-linked autism spectrum disorder, has provided one of the first causal links between microglial alterations and psychiatric disturbances: the authors succeeded in the mitigation of Rett syndrome-like symptoms by supplying wild-type microglia through bone marrow transplantation or genetic rescue in MECP2-null mice (Derecki et al., 2012).

A role for microglia in anxiety and depression is also emerging on the basis of several observations. First, IL-1β and TNF, which are proinflammatory cytokines released during peripheral infection as well as by activated microglia, can induce sickness behavior, resulting in decreased motor activity, fatigue, reduced food intake, anhedonia and social withdrawal in both rodents and humans. Many of these symptoms are commonly observed in depressed patients (Dantzer et al., 2008), whose levels of proinflammatory cytokines have been found increased in both peripheral blood and cerebrospinal fluid (CSF; Zorrilla et al., 2001). Second, *postmortem* studies have revealed consistent microgliosis in suicide-attempters compared to controls (Steiner et al., 2008). Moreover, both activation state of microglia and proinflammatory cytokine levels have been suggested to predict depression relapse and, even, to evaluate the therapeutic response (Miller et al., 2009; Munkholm et al., 2013; Watkins et al., 2014).

Studies on animal models have provided further evidence for involvement of microglia in the pathogenesis of anxious- and depressive-like behaviors. Rodent paradigms of chronic stress, such as repeated constrain or repeated social defeat, have been reported to induce anxious- and depressive-like symptoms, associated to changes in both microglial activation state and morphology in several brain regions (Tynan et al., 2010; Wohleb et al., 2011; Hinwood et al., 2013). Notably, the treatment of stressed animals with the antibiotic minocycline is able to recover microglial homeostasis together with mood dysfunctions (Hinwood et al., 2013).

Both preclinical and clinical data support the theory of the inflammatory etiology for anxious and depressive behavior, and implicate microglia as possible cellular mediators of these mental disorders (Eyre and Baune, 2012).

## EAE Models Behavioral Symptoms of MS

Although it is reasonable to expect that mood alterations in MS patients are a consequence of their physical progressive disability, the prevalence of anxiety and depression, is generally higher in persons with MS with respect to both the general population (Patten et al., 2003) and patients with other neurological disorders (Schiffer and Babigian, 1984; Schubert and Foliart, 1993; Thielscher et al., 2013). In some cases, psychiatric symptoms may occur at the onset of the disease and independently of physical disability (Haussleiter et al., 2009; Lo Fermo et al., 2010; Suh et al., 2010; Rietberg et al., 2011).

While physical disability is the primary target of pharmacological treatment, mood disorders are currently undervalued and undertreated in clinical practice (Marrie et al., 2009), although they can dramatically worsen the quality of life of MS patients. The scarce attention paid to the psychiatric aspects of MS symptomatology is in part due to the lack of knowledge of their pathological basis. In this respect, studies in animal models of MS may represent the unique opportunity to address this critical issue. Although many of the clinical features of mental illness generally cannot be modeled in rodents, some animal models of depression and anxiety provide reliable and measurable correlate of human behavior and allow the study of their molecular underpinnings. Most of MS clinical and histopathological features are well shaped by EAE, in particular the myelin oligodendrocyte glycoprotein p_35–55_ (MOG_35–55_)-induced “chronic” EAE in C57BL/6 mice (Furlan et al., 2009). In such model different phases of the disease can be distinguished: the pre-symptomatic phase with absence of motor deficits, the acute phase starting from the onset to the peak of clinical symptoms and the following chronic phase, in which motor symptoms become milder. Several studies have reported both anxiety- and depression-like behaviors in all EAE clinical phases in correlation with a number of cellular and molecular players (Table 1).

**Table 1:** **Summary of studies about EAE behavioral syndrome**.

Reference	EAE protocol	Behavioral tests	Time (dpi)	Treatment	Behavioral results	Molecular and cellular link
Pollak et al. (2000)	♀ *SJL/J mice*: adoptive transfer PLP-lymph node cells	Sickness behavior (body weight; food and water intake; sucrose-preference; SI)	Acute phase (5–12 dpi); recovery (13–22 dpi); chronic (23–53 dpi)	None	The onset (acute phase) and recovery of the sickness behavior preceded the onset and recovery of the neurological signs	None
Pollak et al. (2003a)	See Pollak et al. (2000)	Sickness behavior (body weight; food intake; sucrose-preference; SI)	Pre-symptomatic phase; acute phase; recovery	None	See Pollak et al. (2000)	*Onset of the behavioral syndrome*: ↑ brain infiltration ↑ IL-1β and TNF mRNAs ↑ IL1β protein and PGE2 *Behavioral recovery*: ↓ cytokine expression
Pollak et al. (2003b)	♀ *SJL/J mice*: see Pollak et al. (2000); ♀ *C57BL/6 mice*: MOG_35–55_ 300 μg; M. tub. (8 mg/ml); PTX 500 ng.	See Pollak et al. (2003a)	Disease onset	Dexamethasone (3 mg/kg ip); IL-1ra (100 mg/kg i.p.); indomethacin (10 mg/kg s.c.); pentoxifylline (100 mg/kg i.p.); at disease onset and for 2–3 days	*Dexamethasone or IL-1ra or indomethacin*: ↓ behavioral symptoms *Pentoxifylline or TNFR1 (−/−)* No behavioral effects *Pentoxifylline + IL-1ra*: ↓↓ behavioral depression.	None
Peruga et al. (2011)	♀*C57BL/6 mice*: MOG_35–55_ 50 μg; M. tub. (500 μg/ml); PTX 100 ng (mild EAE)	Exploratory and anxiety-like behavior (LDT, OFT, SR); depressive-like behavior (PPI, LH)	Late acute and chronic phase (30–80 dpi)	Amitriptyline (10 mg/kg i.p.) start at 20 dpi and stop 40 dpi	*Mild EAE*: ↑ anxiety-like behavior (LDT and SR) ↑ depressive-like behavior (LH); *Amitriptyline*: ↓ Mild EAE depressive-like behavior (SR)	*Mild EAE (hip)*: ↑ CD3+ infiltrates ↑ **microglia activation** ↑ neuronal loss Behavior correlates with ↑ TNF levels and neuronal loss. *Amitriptyline (hip)*: ↑ norepinephrine levels
Musgrave et al. (2011)	♀ C57BL/6 mice; MOG_35–55_ 50 μg; M. tub. (1 mg/ml); PTX 300 ng	Exploratory and anxiety-like behavior (OFT)	0–35 dpi	Phenelzine (PLZ, 15 mg/kg/day i.p.) starting at 7 dpi and lasted 28 days	*PLZ*: ↑ explorative behavior (from the onset of clinical signs)	*Chronic PLZ treatment*: No reduction of **microgliosis** (scr) ↑ catecholamine (in scr, cb, crb and bs)
Rodrigues et al. (2011)	♀ C57BL/6 mice; MOG_35–55_ 100 μg; M. tub. (ns); PTX 300 ng	Anxiety-like behavior (EPM)	Pre-symptomatic phase (9 dpi)	None	No difference in the EPM performance	None
Haji et al. (2012)	♀ C57BL/6 mice; MOG_35–55_ 300 μg; M. tub. (8 mg/ml); PTX 500 ng	Anxiety-like behavior (OFT, EPM)	Pre-symptomatic phase (7, 9 dpi)	Etanercept (10 μg/μl, 4 week i.c.v. minipump) starting 1 week before immunization	*EAE*: ↑ anxiety-like behavior (OFT, EPM) in EAE mice *Etanercept*: ↓ EAE-anxiety like-behavior	*EAE (str)*:↑ **Microglia activation** ↑ TNF protein ↑ sEPSC duration *Etanercept*: ↓ sEPSC duration *(str)*
Acharjee et al. (2013)	♀ C57BL/6 mice; MOG_35–55_ 100 μg; M. tub. (4 mg/ml); PTX 800 ng	Exploratory and anxiety-like behavior (OFT, EPM); depression-like behavior (TST, FST, SI)	Pre-symptomatic phase (9 dpi)	None	*EAE*: ↑ increased anxiety-like behavior (EPM) ↑ depression-like behavior (TST, FST, SI)	*EAE*: No demyelination, **microglial activation** or astrogliosis ↑ IL-1β and TNF mRNAs in hyp (no in amy or hip) ↑ plasma corticosterone levels
Piras et al. (2013)	♂ C57BL/6 mice; MOG_35–55_ 300 μg; M. tub. (2 mg/ml); PTX 500 ng	Anxiety-like behavior (OFT)	Pre-symptomatic phase (0–8 dpi)	Glatiramer acetate (GA, 150 μg/100 μl, s.c.) every day for 7 days before the immunization	*EAE (2, 4, 6 dpi)*: ↑ (n of squares, central squares and latency to rearing) *GA*: ↓ latency to rearing	*EAE*: ↑ blood circulating T cells (from 4 dpi, before CNS infiltration) = kinetic of T cell entry in the blood and of behavioral changes (OFT) *GA*: ↓ behavioral changes T cell retaining in lymph nodes.
Gentile et al. (2015)	See Haji et al. (2012)	Depression-like (TST, FST) and motivation-based behavior (NB)	Pre-symptomatic phase (9 dpi); Acute phase of mice with mild clinical score and preserved motor skills (20 dpi mild-EAE)	IL-1ra (150 ng/day, 4 week i.c.v. minipump) starting 1 week before immunization	*EAE (9 and 20 dpi)*: ↑ depressive-like behavior (TST, FST) ↓ motivation-based behavior (NB) *IL-1ra* ↓ EAE-behavioral alterations (FST)	*EAE (str)*: ↑ IL-1β mRNA ↑ **microgliosis** ↓ DA release Alteration in DA signaling through DA D1- and D2-like receptors *IL-1ra* ↑ *DA release* ↓ **microgliosis** Recovery of DA signaling

*Abbreviations: EAE, Experimental Autoimmune Encephalomyelitis; dpi, day post immunization; PLP, Proteolipid protein peptide; SI, Social Interaction; IL-1β, interleukin-1β; TNF, Tumor Necrosis Factor; PGE2, Prostaglandin E2; MOG_35–55_, Myelin oligodendrocyte glycoprotein p_35–55_; M. tub., Mycobacterium tuberculosis; PTX, Pertussis toxin; i.p., intraperitoneal injection; IL-1ra, interleukin-1 receptor antagonist; s.c., subcutaneous injection; TNFR1, TNF receptor type 1; LDT, Light Dark Test; OFT, Open Field Test; SR, Startle-Response test; PPI, pre-pulse inhibition; LH, Learned Helplessness test; hip, hippocampus; scr, spinal cord; cb, cerebrum; crb, cerebellum; bs, brainstem; EPM, Elevated Plus Maze; i.c.v., intracerebroventricular; str, striatum; sEPSC, spontaneous excitatory postsynaptic currents; TST, Tail Suspention Test; FST, Forced Swimming Test; hyp, hypothalamus; amy, amygdala; NB, Nest Building; DA, Dopamine*.

Former studies by Pollak and colleagues characterized the so-called “sickness behavior” in EAE mice (Pollak et al., 2000, 2003a,b). All the hallmarks of sickness behavior, observed in acute-phase-EAE mice, were recovered in later phases of the disease, with the exception of the body weight. Notably, such behavior affected EAE mice from the day before the onset of neurological symptoms, demonstrating that emotional changes in EAE are not the mere consequence of motor disability (Pollak et al., 2000). Also, chronic treatment with anti-depressant imipramine prevented body-weight loss in EAE mice (Pollak et al., 2002).

Next, they found that the onset of behavioral syndrome coincided with elevation of TNF and IL-1β in the brain and prostaglandin E2 (PGE2) in the hypothalamus (Pollak et al., 2003a). Interestingly, pro-inflammatory cytokines reached their peak expression in the sickness-behavior phase and decreased along with behavioral recovery, which correlated with worsening of motor symptoms, confirming the hypothesis that cytokines sustain the initial process leading to neurological symptoms but their role dampens with time. Moreover, mice showing sickness behavior but lacking neurological deficits had cerebellar levels of cytokines comparable to motor impaired and behaviorally sick mice, corroborating previous observations (Pollak et al., 2000) and linking for the first time behavioral alterations and inflammation in EAE. However, neither the cellular source of cytokines nor the cytokine levels in EAE mice prior to the behavioral depression were investigated. By using different anti-inflammatory approaches aimed at blocking IL-1β, TNF and PGE2 signaling, they further demonstrated the correlation between inflammation and sickness behavior (Pollak et al., 2003b). Notably, the treatment against TNF signaling was ineffective if administered alone, but when associated to IL-1β antagonist (IL-1ra) improved the effect of IL-1ra on behavior, suggesting a synergistic interaction between the two cytokines.

The picture herein described is limited to sickness behavior, which is a phenotypic trait of depression- and anxiety-like behaviors in both humans and rodents (Dantzer et al., 2008). Several ethological paradigms have been developed to assess such behaviors in rodents and some of them have been used to characterize EAE-linked behavioral syndrome. Peruga and colleagues demonstrated both anxiety- and depressive-like behaviors in mice with mild-EAE phenotype (only with tail weakness) during the acute and chronic phases, through well-established behavioral paradigms requiring regular motor abilities, like open field test (OFT), light-dark test (LDT), startle response test (SR) for anxious-like behavior, pre-pulse inhibition (PPI) and learned helpless test (LH) for depressive-like behavior (Peruga et al., 2011). The emotional changes observed in EAE mice were associated with inflammation (lymphocyte infiltration, microglia activation and TNF expression) in peri-hippocampal regions and a significant and progressive neuronal loss in CA1 hippocampal region. Also, although monoaminergic neurotransmitters were not significantly changed in EAE hippocampus, amytriptiline treatment significantly increased norepinephrine levels and attenuated behavioral response. Musgrave and colleagues also addressed a role for catecholamine in EAE-behavioral syndrome: the anti-depressant phenelzine (PLZ) improved EAE motor disability and behavioral performance in the OFT and corrected altered monoamine levels in several brain areas, without affecting microgliosis in the spinal cord (Musgrave et al., 2011). Therefore, they suggested that the behavioral response to PLZ observed in EAE is likely due to normalization of serotonin levels in ventral horn of spinal cord. Although valid, such interpretation lacks information about microgliosis and inflammation in brain areas more likely involved in mood control.

In a study published in 2012, we showed anxiety-like behavior in pre-symptomatic EAE mice (Haji et al., 2012) by OFT and elevated plus maze (EPM) behavioral tasks. Interestingly, such behavior was associated with strong microglia activation, increased TNF levels and potentiated glutamatergic transmission in the striatum of EAE mice. Preventive intracerebroventricular blockade of TNF had anxiolytic-like effect on EAE mice and normalized glutamatergic transmission. Notably, the striatum is a subcortical area involved in MS and EAE (Bermel et al., 2003; Centonze et al., 2009) as well as in mood control (Báez-Mendoza and Schultz, 2013) and striatal glutamatergic transmission alterations already occurs in pre-symptomatic phase (Centonze et al., 2009), suggesting that synaptic dysregulation in this area may account for EAE behavioral changes.

In contrast, others did not detect any signs of anxiety-like behavior in both pre-symptomatic and acute EAE mice at the EPM (Rodrigues et al., 2011). Conversely, Acharjee and colleagues confirmed our findings and showed for the first time depressive-like behavior in pre-symptomatic EAE mice, through classical paradigms for depressive-like behavior in rodents, the tail suspension (TST) and the forced swimming tests (FST; Acharjee et al., 2013). The authors correlated emotional impairment in EAE mice with increased expression of IL-1β and TNF in the hypothalamus. They did detect neither astro-nor microgliosis in the hypothalamus, the amygdala and the hippocampus of EAE brains, concluding that EAE emotional changes were linked to alteration of hypothalamic-pituitary-adrenal axis (HPA), as already suggested for the acute phase of EAE (Pollak et al., 2003a).

Accordingly, we recently linked EAE depressive-like behavior to striatal IL-1β expression and dopaminergic system alterations in the acute phase (Gentile et al., 2015). By studying mice with mild-EAE phenotype, we demonstrated the occurrence of depressive-like (TST and FST) and motivation-based behaviors (nest building test-NB test) in the acute phase of the disease in correlation with striatal and hippocampal microgliosis. Since IL-1β was expressed by microglia in such areas but the cytokine expression raised significantly in EAE striatum, we hypothesized that IL-1β released by microglia in this area may affect the dopaminergic system thus contributing to EAE depressive-like behavior. Accordingly, the preventive central treatment with IL-1ra corrected emotional changes as well as defective striatal dopaminergic neurotransmission, thus linking inflammation-induced neurotransmission alteration and behavior.

Summarizing, TNF and IL1-β alter EAE behavior likely by affecting catecholamine and glutamatergic neurotransmission in several brain areas, pointing to microglia as possible cellular mediator.

## The Impact of MS Drugs on Behavioral Outcomes: The Example of Glatiramer Acetate and Interferon-1 Beta

Most of the drugs approved for MS therapy are immunomodulatory or immunosuppressive agents, providing to variable extent functional recovery. However, they may have a strong impact on mood control, making necessary *ad hoc* pharmacological interventions. Among the currently available therapeutic approaches for MS treatment, interferon-1β (IFNB) and glatiramer acetate (GA) are first-line disease-modifying drugs. Unfortunately, there is some evidence that IFNB treatment exacerbates depressive symptoms more likely than GA (Pandya and Patten, 2002; Goëb et al., 2003; Arnett and Randolph, 2006), therefore in patients with a history of depression GA treatment is often preferred (Wilken and Sullivan, 2007). However, this issue seems not entirely solved, with some studies reporting no differences between IFNB and GA (Kirzinger et al., 2013) or no significant beneficial effect of GA on mood-related outcomes (Jongen et al., 2010).

**Figure 1:**
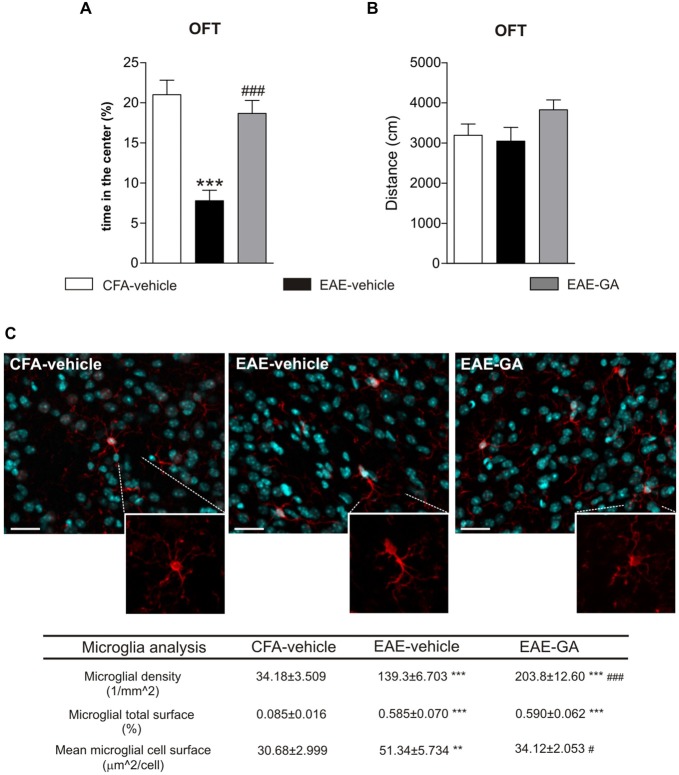
**Glatiramer acetate (GA) treatment protects from EAE-induced anxiety-like behavior and attenuates microglia activation. (A)** Anxiety-like behavior was assessed in pre-symptomatic EAE mice (7 days post immunization, dpi) by means of open field test (OFT). The time in the center of the arena is reduced in EAE-vehicle mice compared to control CFA-vehicle mice and significantly increased in GA-treated mice. CFA-vehicle mice are the experimental control of EAE, receiving the same treatment of EAE mice with the exception of the immunogen MOG_35–55_. Data are expressed as mean ± S.E.M. One-way ANOVA analysis, Tukey’s *post hoc* test: EAE-GA vs. EAE-vehicle ^###^*p* < 0.001, EAE-vehicle vs. CFA-vehicle ***p < 0.001. **(B)** The total distance traveled in the arena was unchanged among the groups, confirming the absence of motor dysfunction in 7 dpi EAE mice. Data are expressed as mean ± S.E.M. One-way ANOVA analysis, Tukey’s *post hoc* test: EAE-vehicle vs. CFA-vehicle *p* > 0.05; EAE-GA vs. EAE-vehicle *p* > 0.05; CFA-vehicle vs. EAE-GA *p* > 0.05. **(C)** GA treatment affects microglia proliferation and activation in the striatum of pre-symptomatic (7 dpi) EAE mice. The microphotographs are low magnification confocal images showing microglial cell density in the striatum of control CFA-vehicle, EAE-vehicle and EAE-GA mice: Iba1 staining for microglia (red; counter-staining with DAPI–blue), reveals increase in microglial cell density in EAE-vehicle vs. CFA-vehicle striatum with further enhancement in EAE-GA striatum. Insets show different morphologies of microglial cells among the groups: microglia from EAE striatum is visibly hypertrophic if compared to both microglia from CFA-vehicle and EAE-GA striatum. Scale bar: 25 μm. Quantitative and qualitative analysis of microglia, based on IBA1 immunofluorescence, is reported down the immunofluorescence images: GA increases microglia density and restores resting state of microglial cells. The morphological analysis of microglial cells, based on the area covered by IBA1 positive cells inside the striatum, shows the effect of GA in reducing microglial hypertrophy observed in EAE-vehicle, expressed as mean cell area, while the total microglial area is similar to EAE-vehicle microglia. For image acquisition and analysis method, refer to Gentile et al., 2013. Data are expressed as mean ± S.E.M. One-way ANOVA analysis, Tukey’s *post hoc* test: EAE-vehicle and EAE-GA vs. CFA-vehicle: ***p < 0.001, **p < 0.01; EAE-GA vs. EAE-vehicle: ^###^*p* < 0.001, ^#^*p* < 0.05.

Due to the restricted literature about EAE-linked behavioral changes, it is clear that animal studies are far from succeeding in clarifying this matter. Only one paper examined MS drug impact on EAE anxiety-like behavior (Piras et al., 2013; Table 1), correlating EAE emotional changes with time-dependent increased peripheral lymphocytosis. The authors observed that GA attenuated lymphocytosis and behavioral impairment in a very early phase of the disease. Consistently, we found that GA reduced anxiety-like behavior in pre-symptomatic EAE mice (7 dpi) by OFT (Figures 1A,B). We previously demonstrated that GA treatment protected against the TNF-induced synaptotoxic effect on striatal glutamatergic transmission by reducing microgliosis and microglia expression of TNF in the striatum of acute-phase EAE mice (Gentile et al., 2013). Interestingly, activated microglia stimulated with GA *in vitro* mimicked the electrophysiological effect of GA treatment in EAE mice. It has been supposed that GA interacts with microglial surface proteins, involved in microglia activation, like MHC-II complex (Fridkis-Hareli et al., 1997) and P2X7 receptor (Caragnano et al., 2012).

Therefore, we examined GA effect on striatal microglia activation of pre-symptomatic mice. In accordance to our previous findings (Haji et al., 2012), a strong microgliosis was observed in pre-symptomatic EAE striatum (Figure 1C), while GA induced additional proliferation of microglial cells with a resting phenotype, suggesting reduced inflammation. The microglial changes induced by GA may abolish the potentiated glutamatergic transmission in the pre-symptomatic striatum, through mechanisms similar to those described in the acute phase. Also, in GA striatum we could occasionally observe amoeboid-like microglial cells, resembling cells in phagocytic activity (not shown): *in vitro* studies on primary murine microglia and human monocytes have shown that GA promotes phagocytosis in those cells (Pul et al., 2011, 2012), likely with protective effects.

Lastly, PET studies in MS patients treated with GA corroborate the effect of GA on microglial activation (Ratchford et al., 2012).

## Conclusions and Perspectives

The investigation of the role of microglia in MS pathogenesis is flourishing, but the contribution of these cells to MS mood disturbances has been only partially addressed. The analysis of pre-symptomatic or non-disabled EAE mice allows dissecting the different contribution of microglia to behavior and motor symptoms.

From the above overview, several elements have emerged: inflammation and microglia activation are involved in EAE-behavioral syndrome and impaired neurotransmission is likely the final outcome of the overactive microglia-immune-neuronal interaction, leading to behavioral alterations. The interplay between microglia and neurons is still poorly explored, but promising and therapeutically attractive. Therefore, we propose to further study the interaction between microglia activation and neurotransmission in brain areas involved in mood control: for example by investigating microglia contribution to EAE/MS hippocampal synaptic dysfunctions (Dutta et al., 2013; Nisticò et al., 2013; Michailidou et al., 2015), and by extending studies in EAE striatum from glutamate and catecholamine to other compromised neurotransmitter systems, such as GABA or cannabinoids (Musumeci et al., 2011; Rossi et al., 2011; Musella et al., 2014). Targeting microglia to limit synaptic damage and behavioral distortion by using immunomodulatory agents, such as GA, or the antibiotic minocycline, known to reduce microgliosis and depressive symptoms (Hinwood et al., 2013), may represent an alternative therapeutic strategy to conventional anti-depressants.

The study of microglia should be fostered in experimental as well as in human investigations and with regard to mood etiology, by using novel *in vivo* imaging techniques. Interestingly, the recent introduction of second-generation PET radioligands has been found able to reveal the extent of microglial activation by quantifying the increased expression of the 18-kDa translocator protein (TSPO) in EAE (Mattner et al., 2013) and MS (Giannetti et al., 2014, 2015) brains. The use of this technique is not yet widespread for economic and safety reasons, but it is viewed as highly promising for EAE/MS pathogenesis studies (Politis et al., 2012b), as well as to monitor and to distinguish drug effects on microgliosis (Airas et al., 2015) and to predict clinical outcomes (Politis et al., 2012a). Increased TSPO binding was observed in normal-appearing gray and white matters in MS remitting patients (Versijpt et al., 2005), corroborating the hypothesis that microglia activation occurs early in the disease and can affect the neuronal compartment (Centonze et al., 2010). The application of such technique to behavioral disorders is embryonic (Kenk et al., 2015): microglial metabolite PET measurements could be correlated to data from psychological assessment in MS or behavioral testing and electrophysiological recordings in EAE.

To conclude, we are confident that the integration of data from clinical and preclinical studies and the combined use of different techniques for monitoring microglia state and action are a fruitful strategy to find novel diagnostic and therapeutic tools for MS mood-related disorders.

## Conflict of Interest Statement

The authors declare that the research was conducted in the absence of any commercial or financial relationships that could be construed as a potential conflict of interest.
